# Mirror Visual Feedback Induces M1 Excitability by Disengaging Functional Connections of Perceptuo-Motor-Attentional Processes during Asynchronous Bimanual Movement: A Magnetoencephalographic Study

**DOI:** 10.3390/brainsci11081092

**Published:** 2021-08-20

**Authors:** Szu-Hung Lin, Chia-Hsiung Cheng, Ching-Yi Wu, Chien-Ting Liu, Chia-Ling Chen, Yu-Wei Hsieh

**Affiliations:** 1 Department of Psychology, Soochow University, Taipei 11102, Taiwan; d99227105@ntu.edu.tw; 2 Department of Occupational Therapy, Graduate Institute of Behavioral Sciences, College of Medicine, Chang Gung University, Taoyuan 33302, Taiwan; ch.cheng@mail.cgu.edu.tw (C.-H.C.); ywhsieh@mail.cgu.edu.tw (Y.-W.H.); 3 Healthy Aging Research Center, Chang Gung University, Taoyuan 33302, Taiwan; 4 Laboratory of Brain Imaging and Neural Dynamics (BIND Lab.), Chang Gung University, Taoyuan 33302, Taiwan; 5 Department of Psychiatry, Chang Gung Memorial Hospital, Linkou, Taoyuan 33305, Taiwan; 6 Department of Physical Medicine and Rehabilitation, Chang Gung Memorial Hospital, Linkou, Taoyuan 33305, Taiwan; clingchen@gmail.com; 7 Department of Physical Medicine and Rehabilitation, Taipei Tzu Chi Hospital, Buddhist Tzu Chi Medical Foundation, New Taipei 231405, Taiwan; ctliu242@gmail.com; 8 School of Medicine, Tzu Chi University, Hualien 97004, Taiwan; 9 Graduate Institute of Early Intervention, College of Medicine, Chang Gung University, Taoyuan 33302, Taiwan

**Keywords:** mirror visual feedback (MVF), motor cortex, beta rebound oscillation, functional connectivity, magnetoencephalography (MEG)

## Abstract

Mirror visual feedback (MVF) has been shown to increase the excitability of the primary motor cortex (M1) during asynchronous bimanual movement. However, the functional networks underlying this process remain unclear. We recruited 16 healthy volunteers to perform asynchronous bimanual movement, that is, their left hand performed partial range of movement while their right hand performed normal full range of movement. Their ongoing brain activities were recorded by whole-head magnetoencephalography during the movement. Participants were required to keep both hands stationary in the control condition. In the other two conditions, participants were required to perform asynchronous bimanual movement with MVF (Asy_M) and without MVF (Asy_w/oM). Greater M1 excitability was found under Asy_M than under Asy_w/oM. More importantly, when receiving MVF, the visual cortex reduced its functional connection to brain regions associated with perceptuo-motor-attentional process (i.e., M1, superior temporal gyrus, and dorsolateral prefrontal cortex). This is the first study to demonstrate a global functional network of MVF during asynchronous bimanual movement, providing a foundation for future research to examine the neural mechanisms of mirror illusion in motor control.

## 1. Introduction

Mirror visual feedback (MVF) during practice of a hand motor skill was shown to improve the performance of both hands [1]. The fact that MVF leads to behavioral gains in hand movements has been applied in neurorehabilitation regimens such as mirror therapy (MT) for recovering motor impairment of the upper limb in stroke patients [2,3]. In MT, patients are asked to synchronously perform bilateral hand movements while observing the MVF of their unaffected upper limb as if it were the affected limb. MT is evidenced to have moderate effects on improving upper limb performance [4]. Understanding the underlying mechanisms of MT, including the modulation of MT on brain activities, might help health professions design dedicated programs to augment MT effects [5].

Various image studies recruiting healthy participants or stroke patients demonstrated that MVF of the unmasked moving hand, without the movement of the masked hand, can increase the activity of primary motor cortex (M1) ipsilateral to the unmasked moving hand [1,6,7,8,9]. Instead of the enhancement in M1 excitability, other image studies using positron emission tomography and functional magnetic resonance imaging (MRI) showed that MVF induces activations of various brain regions other than motor-related ones. Specifically, the activation of the primary visual cortex (V1) ipsilateral to the moving hand was noted during only unmasked hand movement with a mirror [10,11]. The superior temporal gyrus (STG) and superior occipital gyrus that were responsible for perceptuo-motor coordination, rather than motor or premotor areas, were activated by the MVF of hand movements [12]. The dorsolateral prefrontal cortex (DLPFC) is a higher-order region responsible for visual attention, especially for maintaining action in case of a conflict between the proprioception of actual movement and the perceived movement of visual feedback [13]. For example, the participant performed asynchronous movement of hands that were unmasked and masked, and perceived synchronous visual feedback due to the mirror reflection.

These aforementioned studies exclusively looked at the activation of brain areas induced by MVF. Very limited studies underscore the possible connectivity of different regions during performing movement with MVF, and these studies mostly focused on the functional connections among sensorimotor regions [14,15]. According to the findings of previous neuroimaging studies, MVF may induce M1 excitability by modulating the functional connections among brain regions associated with visual perception (i.e., V1), perceptual-motor coordination (i.e., STG), motor execution (i.e., M1), and attentional process (i.e., DLPFC) [16]. No study to date has explored the connectivity of these brain areas involving the whole process of MVF tasks. Understanding the underlying functional networks that respond to MVF will provide a neurophysiological basis for clinical application, such as identifying other target regions for noninvasive brain stimulation techniques.

Instead of MRI, which specializes in high spatial resolution, magnetoencephalography (MEG) takes advantage of excellent temporal resolution (better than 1 ms) and reasonable spatial resolution that have been aided by recent advances in computing algorithms and hardware. MEG is a brain imaging technique that directly measures cortical neural activity at different frequencies (termed oscillations), and unlike the blood oxygenation level dependent signal used by functional MRI, it is unaffected by the effects of neurovascular uncoupling. Moreover, a distributed source modeling method (e.g., minimum norm estimate) used in MEG studies has made MEG a suitable device for clarifying the changes in regional activation and network connectivity at the source level [17,18].

Although MEG has the advantages mentioned above, few studies have used MEG to underscore the mechanisms of MVF [16,19]. These studies usually recruited healthy participants and used an electricity-induced beta rebound oscillation (~20 Hz) as an indicator of the M1’s functional status to monitor the changes in M1 activation [8,9,20,21]. In addition, the beta-band oscillations in the sensorimotor cortex tend to be associated with processing or preparing to process movement execution [22,23,24].

In addition to beta oscillations, various MEG and electroencephalography (EEG) studies considered alpha-band oscillation (~8–12 Hz) as a local marker indicating the somatosensory and visual information process as well as the function of attention [22,25,26,27]. Hence, examining the modulation of alpha- and beta-band oscillations under different functional connections between brain regions that respond to MVF during movement control may shed light on the underlying mechanism of the process.

More specifically, the aims of the present study were two-fold. The first was to use MEG to determine how MVF modifies M1 activation in healthy participants during asynchronous bimanual movement. In this case, we required healthy participants to perform asynchronous bimanual movement in which their left hand performed partial range of movement while their right hand executed full range of movement to indicate normal movement of the unaffected hand. In addition to a control condition (resting condition), we manipulated the presence and absence of the MVF during asynchronous bimanual movement in two separate conditions to differentiate the effects of MVF on the excitability of M1. The second aim was to recognize how the functional connections among different brain regions that respond to the MVF vary between the presence and absence of MVF.

We hypothesized that greater M1 activity should be found when using MVF for asynchronous hand movement compared with the absence of the MVF condition. More importantly, to efficiently enhance M1 excitability, the MVF would reduce brain efforts to engage in the functional networks responsible for the perception, perceptuo-motor, motor, and attentional processes. The results of the current study provide a foundation for future empirical studies to examine the dynamic functional networks in the case of MVF. In addition, clinical researchers can use this functional network to examine the underlying mechanism of MVF in stroke patients and propose useful indicators to determine the efficacy of MT.

## 2. Materials and Methods

### 2.1. Participants

The study recruited 16 right-handed healthy volunteers (8 women). They were a mean age of 23.44 ± 2.28 years (range 20–28 years). Informed written consent was obtained from all the participants, and this consent was approved by the Taipei Veterans General Hospital Institutional Review Board (Taipei, Taiwan). All methods were carried out in accordance with relevant guidelines and regulations.

### 2.2. Stimulation

Each participant’s left median nerve was stimulated at the wrist with 0.2 ms square-wave pulses by a saddle-type electrode during the entire experiment. To avoid expectation effects, the interval between stimulations varied from 1.8 to 2.2 s. We measured the minimum intensity of electrical stimulation sensation (sensory threshold) that each participant could report and used 1.2 times this intensity as the stimulation intensity of each participant. This stimulus intensity of the abductor pollicis brevis is used to obtain the cortical responses with a better signal-to-noise ratio [28,29,30]. Participants were instructed to ignore the stimulation and to focus on the tasks we requested.

### 2.3. Experimental Design and Procedures

**Figure 1:**
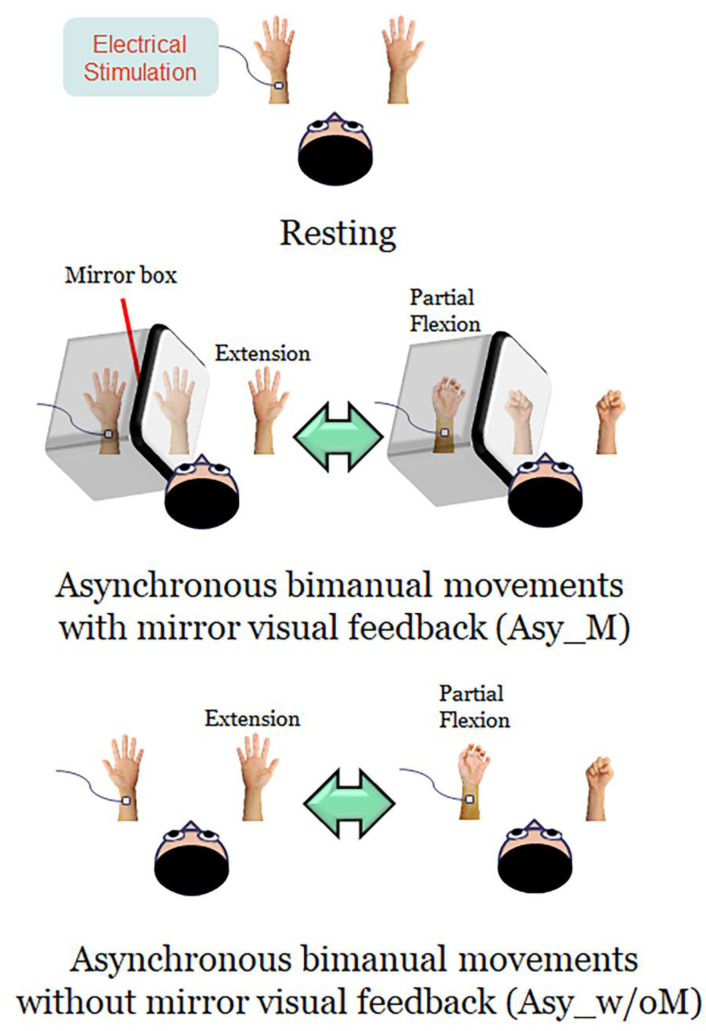
Illustration of the experimental design. In the resting condition, the participants were instructed to look at both stationary hands. In the asynchronous bimanual movement with mirror visual feedback (Asy_M), participants performed bimanual in-phase fingers flexion/extension with a mirror covering the left hand, in which the left hand performed partial range of movement while performing the full range of movement with their right hand. In the asynchronous bimanual movement without the mirror visual feedback (Asy_w/oM), the setup was similar to the Asy_M condition except that the mirror box was removed. During the whole experimental procedure, the left median nerve was stimulated at the wrist to probe the beta rebound oscillations of the primary motor cortex (M1).

Participants comfortably sat upright with their heads resting in the helmet-shaped MEG device (Vectorview, Elekta-Neuromag, Helsinki, Finland). Neuromagnetic responses were recorded in three conditions in a randomized order (Figure 1).

(1) Resting: Both hands were kept stationary with the forearms supinated, and the participants were instructed to look at both stationary hands.
(2) Asynchronous bimanual movement with MVF (Asy_M): The participant’s left hand was masked by a mirror box and the participant was instructed to look at both the unmasked right hand and the mirror reflection of the right hand. Participants were instructed to perform bimanual in-phase fingers flexion/extension repetitively with a frequency of approximately 1 Hz, with their masked left hand performing partial range of movement and their right hand performing full range of movement. The mirror reflection of the right hand is as if the left hand performed the movement the same as the right hand. In this condition, participants performed asynchronous bimanual movement while receiving the visual feedback of synchronous bimanual movement.
(3) Asynchronous bimanual movement without MVF (Asy_w/oM): All settings were similar to the Asy_M condition, except that the mirror box was removed in this condition. Participants were instructed to look at this asynchronous bimanual movement. In this case, participants performed asynchronous bimanual movement and received the visual feedback of asynchronous bimanual movement.

To ensure that participants were familiar with in-phase asynchronous bimanual movement with a constant frequency at approximately 1 Hz, substantial practice of 3 to 5 min was delivered to each participant before the MEG recordings. Throughout the recording process, we used a camera to monitor the performance of the participants to ensure that their movement frequencies are consistent.

### 2.4. MEG Recordings

Neuromagnetic activities were measured continuously during the movement task using a 306-channel MEG (Vectorview, Elekta-Neuromag, Helsinki, Finland) at Taipei Veterans General Hospital. The head position relative to the MEG sensors was registered at the beginning of each block by measuring the magnetic signals produced by current leads to four head position indicators at the forehead (left and right) and bilateral mastoids. The three fiducial points based on a Cartesian coordinate system were determined using a three-dimensional digitizer. The *x*-axis ran from the left to right preauricular points, the *y*-axis passed through nasion, and the *z*-axis pointed in the inferior-superior direction. The MEG data were sampled at 1000 Hz with an online bandpass filter (from 0.1 to 200 Hz) and processed with MaxFilter software (version 2.0) based on the temporal extension of the signal space projection (SSP) algorithm to reduce artifacts originating inside and outside the MEG [31].

### 2.5. Analysis of M1 Beta Rebound Oscillation

The modeling of cortical responses was implemented in Brainstorm software (version 3.4 160620) [32]. At the beginning, we used SSP to remove the artifacts contaminated by eye blinks. The forward problem of MEG measures was resolved by means of the overlapping-sphere method [33]. The depth-weighted minimum norm estimate was used to compute cortically constraint source activation, over ~7500 elementary dipole locations in each hemisphere. The individual source maps were geometrically rescaled to the ICBM152 brain template by Brainstorm’s registration methods.

To calculate the power spectrum, we removed evoked responses from each trial of the raw data (100 ms before and 1000 ms after the stimulus onset), and the data in the identified regions of interest (ROI) were transferred using Morlet-wavelet time-frequency decomposition with a central frequency of 1 Hz and a time resolution of 3 s. The power of signal fluctuations was estimated and exhibited between 1 and 50 Hz in 1 Hz steps.

The mean strength of the most reactive beta oscillation (2 Hz for consecutive bins) in M1, approximately 4 to 5 cm^2^, was identified and calculated from the average of 200 ms centering peak latency of beta rebound oscillations (i.e., 100 ms before and 100 ms after the peak) [28,29,30,34]. The time-resolved magnitude of each elementary dipole was normalized to its fluctuations over the pre-stimulus baseline, yielding a set of *Z*-score time series. We calculated the mean power of the beta rebound oscillation in each condition from all the participants.

The suppression index of beta power in the Asy_M and Asy_w/oM conditions with respect to the resting condition was used to indicate the magnitude of M1 activation: [suppression index = (β_resting_ − β_Asy_M or Asy_w/oM_)/β_resting_ × 100%]. A suppression index with a larger value suggests a higher M1 activation.

### 2.6. Analysis of Functional Connectivity

**Figure 2:**
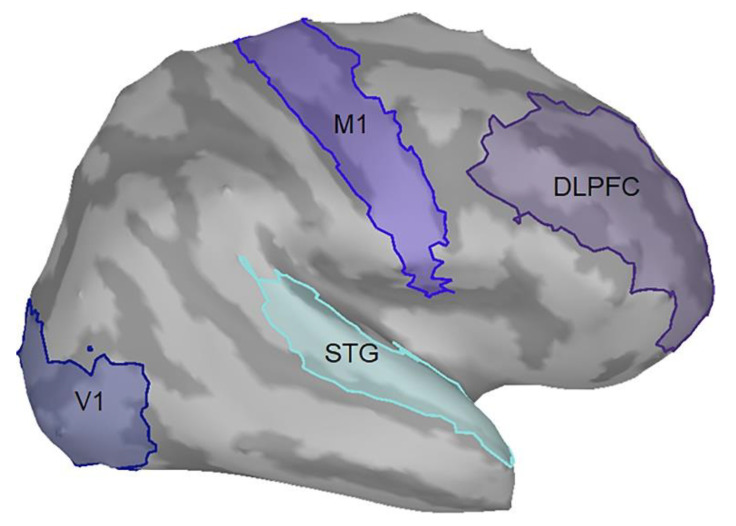
Selection of regions of interest on the ICBM152 cortical surface. DLPFC, dorsolateral prefrontal cortex; V1, primary visual cortex; M1, primary motor cortex; STG, superior temporal gyrus.

In the current study, we were particularly interested in how MVF modulates M1 excitability through the functional networks among brain regions across perceptual, motor, and attentional processes; therefore, we identified four brain areas based on the Desikan-Killiany Altas of ICBM152 brain template: V1 (visual perception), STG (perceptuo-motor coordination), M1 (motor execution), and DLPFC (attentional process) as the ROIs. Each ROI contains multiple vertices (i.e., dipoles); we used the averaged value of the vertices to perform functional connectivity analysis. These brain regions were primarily considered to be activated or functionally connected, or both, with other regions while implementing the upper limb movement with MVF according to the literature (Figure 2) [12,13,16,35]. The procedures of time-frequency decomposition were the same as those aforementioned. The entire epoch (i.e., 1000 ms) of each raw trial was used to compute the functional connectivity. The source-based coherence among V1, STG, DLPFC, and M1 was estimated by using magnitude-squared measures with a maximum frequency resolution of 1 Hz and the highest frequency of interest of 50 Hz.

Coherence is one mathematical method for quantifying frequency-dependent correlations of brain activity measured by two or more brain regions. The coherence strength can be used to determine whether two or more brain regions have similar neuronal oscillatory activity with each other. The oscillatory activities of these coherence measures were classified and averaged into alpha (8 to 12 Hz) and beta (13 to 30 Hz) bands.

### 2.7. Statistical Analysis

The data are presented as mean ± standard error of the mean. To avoid increasing vulnerability to the assumption violations of parametric analysis and type I errors, the suppression index of beta power and functional connectivity differences between two experimental conditions (Asy_M and Asy_w/oM) were evaluated by nonparametric Wilcoxon signed-rank tests for estimating *p* values (one-tailed). Statistical significance was set at *p* < 0.05. For the analysis of functional connectivity differences, the reasons for comparing six different coherence pairs for each participant in two different frequency bands for two different conditions, but not all factors in one model, for analysis are described below. Depending on numerous previous findings, each pair of brain regions and each frequency band may represent different functional processes during motor control with MVF [16]. Furthermore, we were interested in recognizing how the functional connection among different brain regions operates differently between two experimental conditions, rather than differences between different pairs of brain regions and different frequency bands. Integrating all factors into one model to analyze these effects could have resulted in ignoring small but valuable differential effect between the two experimental conditions.

## 3. Results

### 3.1. Analysis of M1 Beta Rebound Oscillation

**Figure 3:**
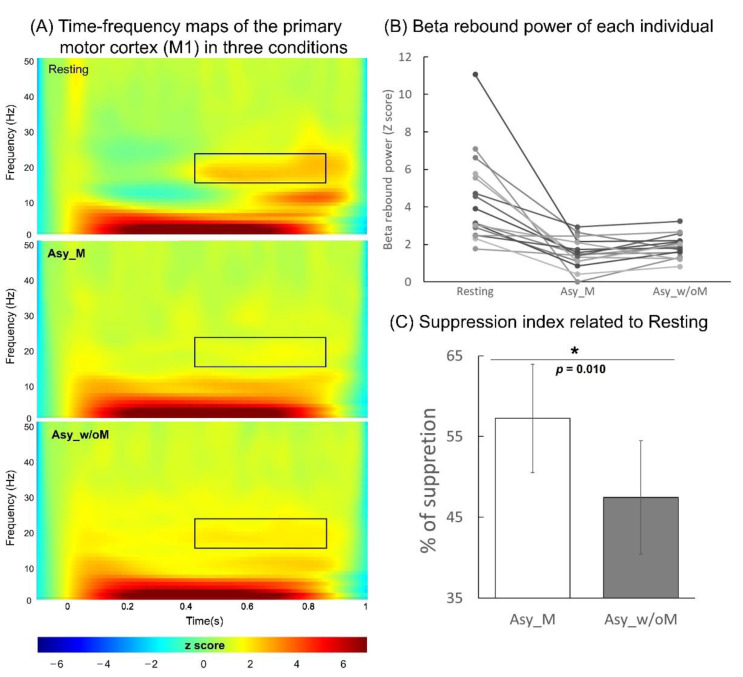
(**A**) Grand-averaged time-frequency maps of electricity-induced beta rebound oscillation (black rectangles) of the right primary motor cortex (M1) in the resting, asynchronous bimanual movement with mirror visual feedback (Asy_M), and asynchronous bimanual movement without mirror visual feedback (Asy_w/oM) conditions. (**B**) The M1 beta rebound strength from each individual in the three conditions. (**C**) The suppression index refers to the extent of beta power suppression in two manipulated conditions with respect to the resting condition. The beta rebound oscillations were suppressed more under Asy_M than under Asy_w/oM.

The grand-averaged time-frequency maps at the right M1 of the three conditions are displayed in Figure 3A: the time interval was −100 to 1000 ms and the frequency band was 1 to 50 Hz. In the resting condition, the electricity-induced beta oscillations were immediately decreased after the stimulation of the left median nerve and then rebounded above the baseline level before the stimulation within the time window of 400 to 900 ms. To indicate the mean power of the beta rebound oscillations in each condition, we used the peak latency of the oscillations within this time window from all of the participants, and calculated from the average of 100 ms before and 100 ms after the peak latency of the oscillations. The results of the mean power of the beta rebound oscillations for each participant are shown in Figure 3B, indicating that the Asy_M and Asy_w/oM conditions were substantially reduced compared to the resting condition. Specifically, the statistical results of the suppression index indicated that the suppression effect on the beta rebound oscillations were greater under Asy_M than under Asy_w/oM (*Z* = −2.28, *p* = 0.010; Figure 3C). These results demonstrated that when executing MVF to perform asynchronous bimanual movement, the M1 was activated more.

### 3.2. Analysis of Functional Connectivity

**Figure 4:**
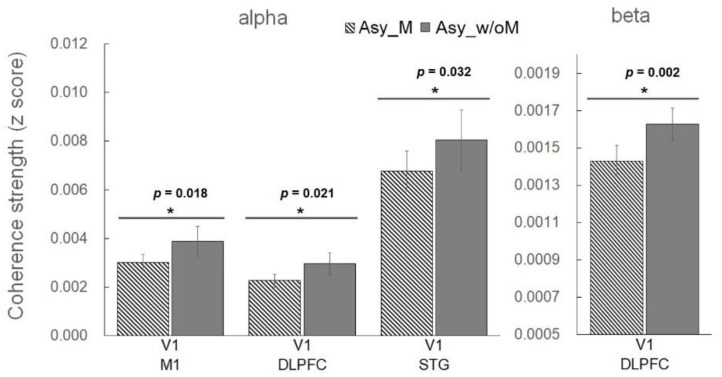
The statistical results show that functional connectivity of V1-M1 (alpha band), V1-DLPFC (both alpha and beta bands), and V1-STG (alpha band) in the Asy_M condition was significantly reduced compared with the Asy_w/oM condition.

We also evaluated the effects of MVF on the coherence strength among the V1, STG, DLPFC, and M1 regions across all the participants for the alpha and beta bands. Figure 4 shows the significant comparisons of coherence strength between the Asy_M and Asy_w/oM conditions. For the comparisons in the alpha band, the cortical coherence between V1 and M1 (V1-M1), V1 and STG (V1-STG), and V1 and DLPFC (V1-DLPFC) was significantly smaller under Asy_ M than under Asy_w/oM (*Z* = −2.04, *p* = 0.018; *Z* = −1.82, *p* = 0.032; *Z* = −1.99, *p* = 0.021). In the beta band, the coherence strength between the V1 and DLPFC (V1-DLPFC) was smaller under Asy_M than under Asy_w/oM (*Z* = −2.29, *p* = 0.002), whereas the results for the other comparisons were not statistically significant (*p* > 0.06).

## 4. Discussion

We required the participants in this study to perform asynchronous bimanual movement (i.e., partial range of movement behind the mirror), and identified the functional connections among four brain regions (V1, STG, DLPFC, and M1) to study the influences of MVF on modulating the activity of M1 ipsilateral to the unmasked hand. The suppression index results indicated that the beta rebound oscillations were significantly more suppressed under Asy_M than under Asy_w/oM (*Z* = −2.28, *p* = 0.010), demonstrating that M1 was activated more when performing asynchronous bimanual movement with MVF. To realize the neuromodulatory effects associated with MVF on M1 excitability, we compared the functional connectivity patterns between Asy_M and Asy_w/oM conditions. The functional connectivity of V1-M1 and V1-STG in the alpha band as well as V1-DLPFC in both the alpha and beta bands showed less coherence strength under Asy_M than under Asy_w/oM. These findings may indicate that the MVF reduced the engagement of distributed networks within the brain, including regions related to perceptual [12,36], motor [13,16,20,37], and attentional process [38,39], to efficiently enhance M1 excitability.

Consistent with previous findings, beta rebound oscillations were reduced in the M1 when performing hand movements compared with the resting state, indicating enhanced M1 activities during hand movements [6,10,11,40,41,42]. M1 excitability was greater in the presence of MVF compared with the absence of MVF, considering beta rebound oscillations under Asy_M were greater than under Asy_w/oM as expressed by the suppression index.

As shown by results of coherence strength in Figure 4, the coherence strength of V1 and other specified brain regions was reduced significantly under Asy_M compared with the strength under Asy_w/oM either in the alpha band (V1-M1, V1-DLPFC, and V1-STG) or in the beta band (V1-DLPFC). These findings indicate that M1 activity modulated by MVF should be associated with reduced synchronization of the functional networks among visual perception (V1) with motor execution (M1), perceptuo-motor coordination (STG), and attentional process (DLPFC).

Our results demonstrate that the coherence strength of V1-M1 and V1-STG in alpha-band oscillations was reduced under Asy_M compared with Asy_w/oM. The alpha-band oscillations are considered to be closely linked to somatosensory and visual processing [22,25,27]. The Asy_M condition involved inconsistency between the visual input from the mirror illusion of the full range of hand movement and proprioceptive/kinesthesia feedback from the partial range of hand movement. This inconsistency might explain the results of less coherence of visual-related neural activity and motor-/perceptuo-motor-related neural activities. The activation of visual neurons is less synergistic with the activations of motor and perceptuo-motor neurons under Asy_M than under Asy_w/oM, which might leave more resources for M1 activation under the former condition. In contrast, the coherence strength of V1-M1 showed similar intensity in the beta band under the two conditions. It might be that oscillations of the beta band tend to be related to hand movement production and that both conditions involved the same asynchronous movement patterns [8,22,43]. Accordingly, no difference in the coherence strength between two conditions was found. The finding of the difference between the two conditions suggests that the alpha-band oscillations can be a plausible indicator to demonstrate the visual-motor and visuo-perceptuo-motor neural networks during asynchronous bimanual movement during MVF.

We found that the coherence strengths of V1-DLPFC in both alpha- and beta-band oscillations were reduced when receiving MVF during bimanual movement. The findings suggest that MVF helps to lower attention demands toward not only perceptual process of visuo-motor conflict but also asynchronous movement output represented by the alpha and beta bands, respectively. To the best of our knowledge, this is the first study to demonstrate beta-band oscillations of the visuo-attention process modulated by MVF. Future research may investigate the role of these oscillations in the interaction of these visual perception and attentional processes.

Although we showed a correlation within and between different functional networks (perceptual, motor, perceptuo-motor, and attentional process) across four brain regions (V1, DLPFC, STG, and M1) located in various brain areas, the causal relations between these functional networks remain unclear. Hence, future studies that use noninvasive brain stimulation to selectively target these functional networks to investigate causal relationships among these functional networks are encouraged. Furthermore, we used healthy individuals to simulate the hand movements of stroke patients. The present study findings will need to be validated in clinical populations such as those recovering from stroke for clinical application.

## 5. Conclusions

To the best of our knowledge, this study is the first to investigate the effects of MVF on the functional connectivity among broad brain regions associated with visual perception, perceptuo-motor coordination, motor, and attention processes during asynchronous bimanual movement. We demonstrated that M1 activity is increased and alleviated the redundant neural correlates between V1 and other brain regions associated with perceptuo-motor coordination (STG), motor (M1), and attention (DLPFC) processes by receiving MVF during asynchronous bimanual movement. These findings are consistent with known motor learning principles that attribute the success of motor implementation to visuo-spatial perceptual focus and attentional processing. MVF affects multiple functional networks, suggesting it can serve as a versatile tool to promote motor learning or recovery.

This study provides a neurophysiological basis for explaining the possible neural mechanisms of MVF often used in MT for the rehabilitation of stroke survivors. Identification of possible target regions beyond M1 responsible for MVF implementation might provide insights into adjuvant techniques (e.g., noninvasive brain stimulation techniques) to reduce the activations of these areas to promote M1 activities and motor recovery during MT in stroke survivors. Further research that includes stroke survivors is warranted to fully understand the causal relationship among these functional networks during MT and possible augmented effects of combining noninvasive brain stimulation with MT.

## Data Availability

The data presented in this study are available on request from the corresponding author. The data are not publicly available due to their confidentiality.
